# Concurrent administration of trastuzumab and anthracyclines as adjuvant regimen for HER2-positive breast cancer: a randomised controlled trial

**DOI:** 10.18632/oncotarget.21579

**Published:** 2017-10-06

**Authors:** Songjie Shen, Ying Xu, Yidong Zhou, Feng Mao, Jinghong Guan, Qiang Sun

**Affiliations:** ^1^ Department of Breast Surgery, Peking Union Medical College Hospital, Peking Union Medical College, Chinese Academy of Medical Sciences, 1 Shuaifuyuan, Dongcheng District, Beijing 100730, China

**Keywords:** breast cancer, adjuvant therapy, trastuzumab, anthracycline, cardiac safety

## Abstract

**Background:**

The regimen of concurrent administration of trastuzumab and anthracyclines in the adjuvant treatment of breast cancer has never been evaluated prospectively for fear of cardiac toxicity.

**Methods:**

Patients with HER2-positive operable breast cancer were randomised to receive adjuvant treatment with concurrent or sequential administration of trastuzumab and anthracyclines. Cardiac monitoring was scheduled at baseline and every 3 months after the first dose of trastuzumab. The primary study endpoint was cardiac safety. Secondary endpoints were disease-free and overall survival.

**Results:**

From 2011 to 2014, 201 participants were enrolled and randomised. The median follow-up time was 42 months. Nineteen patients (19.4%) in the concurrent group and 22 patients (22.4%) in the sequential group met the criteria for cardiac events with non-significant difference (*P*=0.598). There was no difference in the mean LVEF between the two groups at the baseline and at 3, 6, 9, 12, and 24 months after the first dose of trastuzumab. No case of congestive heart failure or cardiac death occurred. The differences between the efficacies of the two regimens, defined by disease-free or overall survival, were not significant.

**Conclusions:**

Concurrent administration of trastuzumab and anthracyclines is a safe adjuvant regimen and it provides evidence for further clinical trials.

## INTRODUCTION

The human epidermal growth factor receptor 2 (HER2) is overexpressed in approximately 15%–25% of invasive breast cancers, and is associated with a high risk of disease recurrence and reduced survival [1–3]. Trastuzumab, a monoclonal antibody directed against HER2, has been shown to improve disease-free survival (DFS) and overall survival (OS) in HER2-positive breast cancer patients [1–3]. Moreover, many clinical trials have also indicated that patients with HER2-positive breast cancer might derive preferential benefit from anthracyclines [4–6].

However, cardiac dysfunction is the most concerning toxicity associated with these regimens, especially when trastuzumab is given concurrently with anthracyclines. In a pivotal trial in the metastatic setting, the concurrent administration of trastuzumab and anthracyclines was reported to result in unacceptably high rates of cardiac dysfunction (27%), although the highest survival benefit was also observed in this treatment [7]. However, in the neoadjuvant therapy, trastuzumab administered concurrently with anthracyclines has shown exceedingly low cardiotoxicity [8–11], and expectedly high rates of pathological complete response (pCR) and prolonged survival [8–10].

Although the findings of the North Central Cancer Treatment Group N9831 trial indicated an increase in disease-free survival with concurrent trastuzumab and paclitaxel compared to sequential regimen in the adjuvant treatment of breast cancer [12], only the sequential regiment of trastuzumab and anthracyclines administration was evaluated in prospective clinical trials. To date, the safety and efficacy of trastuzumab administered concurrently with anthracyclines has never been evaluated prospectively in the adjuvant treatment. Concurrent administration of trastuzumab and anthracyclines was not recommended in the adjuvant therapy of breast cancer in all the guidelines, including the latest version of the National Comprehensive Cancer Network (NCCN) guidelines of breast cancer (version 2.2016) and the American Society of Clinical Oncology (ASCO) guideline adaptation of the Cancer Care Ontario clinical practice guideline [13], because fear of cardiac toxicity. Thus, we conducted the present prospective, randomised, and controlled trial to assess the cardiac safety, as well as the therapeutic efficacy, of the concurrent administration of trastuzumab and anthracyclines in the adjuvant treatment of HER2-positive early breast cancer.

## RESULTS

### Patient characteristics

**Figure 1:**
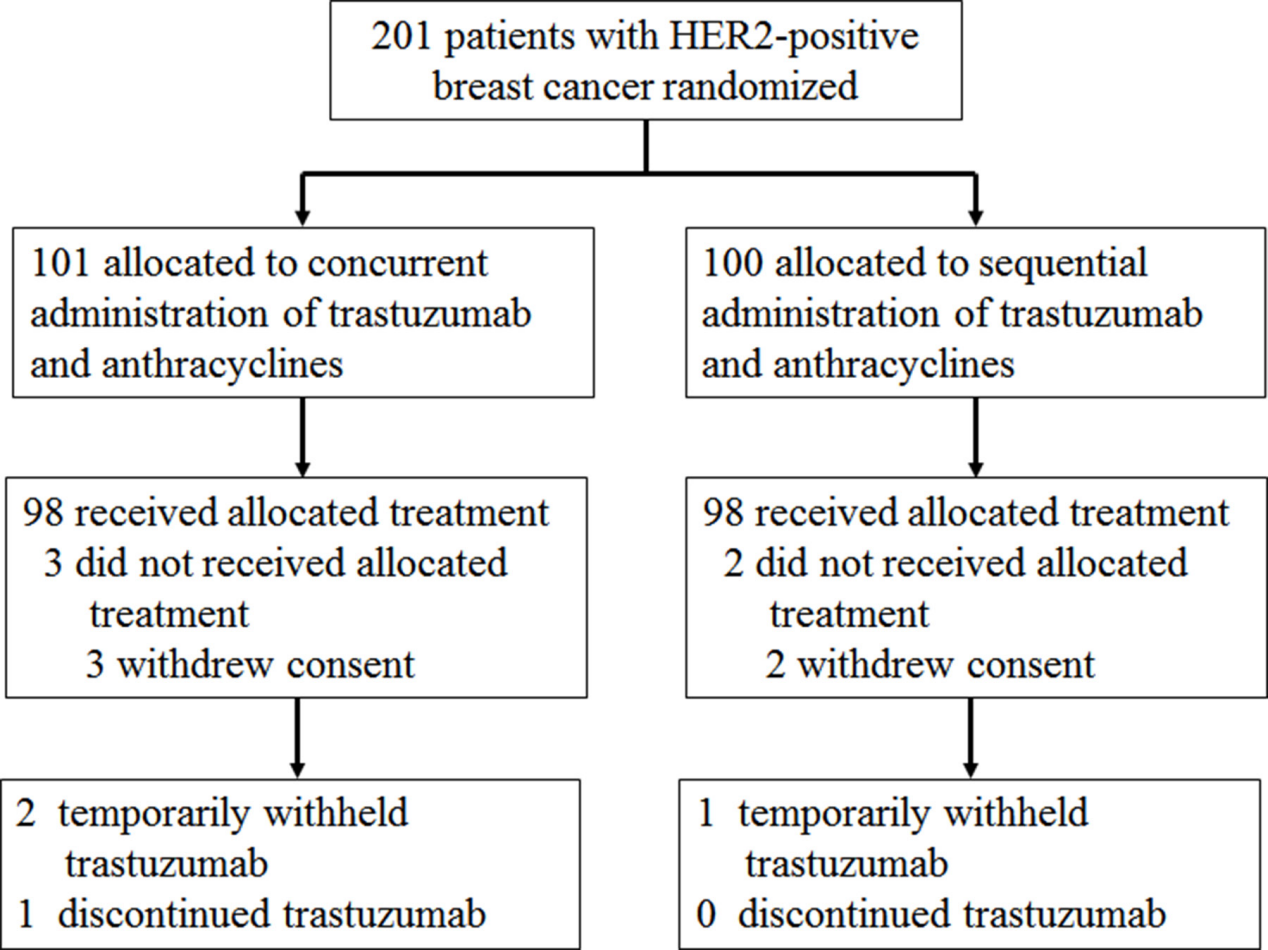
CONSORT diagram HER2, human epidermal growth factor receptor.

A total of 201 patients were enrolled (101 in the concurrent group and 100 in the sequential group) in the investigation from August 10, 2011 to March 1, 2014 (Figure 1). The last follow-up date was August 1, 2016, and the median follow-up time was 42 months (range: 19–62 months). Three of these patients in the concurrent group and two participants in the sequential group withdrew consent before starting treatment. Therefore, 196 participants (98 in the concurrent group and 98 in the sequential group) were eligible for final analysis. Table 1 showed the baseline characteristics for the concurrent and sequential groups. Characteristics were well balanced between the two treatment groups.

**Table 1:** Demographic and baseline characteristics

Variable, N (%)	Concurrent group(n=98)	Sequential group(n=98)	*P*-value
Age(years)			
Mean±SD	48.0±9.0	48.7±9.5	0.628
Median (range)	49.5 (25∼67)	50.0 (28∼66)	
Age Group			0.986
≤35	10	10	
36∼55	64	63	
>55	24	25	
BSA, m^2^	1.73	1.74	
Mean±SD	1.72±0.12	1.74±0.11	0.499
Median (range)	1.71 (1.43∼2.01)	1.72 (1.45∼2.06)	
History of hypertension	14	12	0.674
Tumor size (cm)			0.907
≤2.0	48	51	
2.1∼5.0	45	42	
>5.0	5	5	
Grade			0.827
G1	5	4	
G2	44	48	
G3	49	46	
ER positive	47	52	0.475
PR positive	39	42	0.663
p53 positive	56	63	0.306
Ki-67 high(≥14%)	81	76	0.371
Lymph nodes			0.867
0	38	37	
1∼3	25	28	
4∼9	13	15	
>9	22	18	
AJCC stage			0.839
I	24	23	
II	38	42	
III	36	33	
Chemotherapy Regimen			0.539
Anthracyclines, no taxanes	33	29	
Anthracyclines plus taxanes	65	69	0.059
Concurrent	37	28	
Sequential	28	41	
Cumulative anthracyclines dose (mg/m^2^)	361.2	359.1	0.869
Radiotherapy	43	48	0.474
Left-sided tumor	24	23	
Endocrine Therapy	51	55	0.566

SD, standard deviation; BSA, body surface area; ER, estrogen receptor; PR, progesterone receptor.

### Cardiac safety and other safety

**Figure 2:**
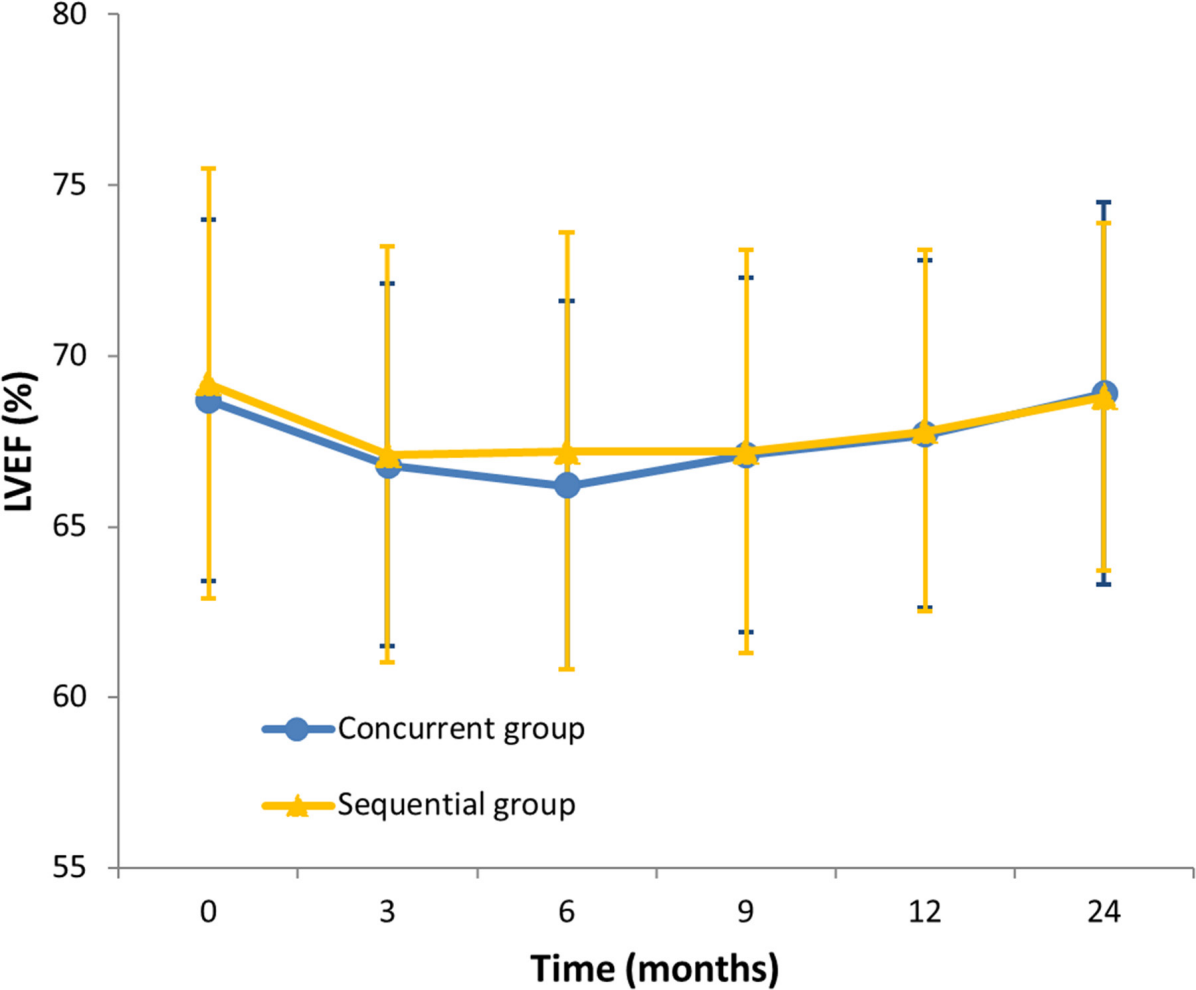
Mean left ventricular ejection fraction (LVEF) measurements during treatment and follow-up Vertical bars represent standard deviation.

The cardiac safety events were graded according to the NCI-CTC, version 2.0. Trastuzumab and anthracyclines was well tolerated in both the concurrent group and the sequential group. There was no patient documented congestive heart failure or cardiac death. The primary cardiac safety event in both treatment groups was an asymptomatic decrease in the left ventricular ejection fraction. Nineteen patients (19.4%, 95% CI 12.4-28.9) in the concurrent group and 22 patients (22.4%, 95% CI 14.9-32.2) in the sequential group met the criteria for cardiac events (odds ratio 0.831, 95% CI 0.417-1.656, *P* = 0.598, Table 2). No difference between the two groups was found in the mean LVEF determined at baseline, every 3 months during trastuzumab administration, and at 24 months after the first dose of trastuzumab (Figure 2). In the concurrent group, more than 10% but less than 20% reduction in LVEF (NCI-CTC Grade 1) was detected in 17 patients (17.3%, 95% CI 10.7-26.6), whereas a reduction higher than 20% in LVEF or a decline below 50% (NCI-CTC Grade 2) was established in two patients (2.0%, 95% CI 0.3-7.9). On the other hand, in the sequential group, 21 patients (21.4%, 95% CI 14.0-31.1) exhibited a Grade 1 LVEF reduction, and 1 patient (1.0%, 95% CI 0.1-6.4) manifested a Grade 2 reduction. Trastuzumab was temporarily withheld in two participants in the concurrent group and one patient in the sequential group with Grade 2 LVEF reduction. The LVEF values of these three patients recovered in four weeks, and trastuzumab administration was continued. Trastuzumab was permanently discontinued in one patient in the concurrent group after the application of 14 doses because of hearing loss.

**Table 2:** Cardiac events during treatment and follow-up

	Concurrent group(N=98)	Sequential group(N=98)	*P*-value
LVEF reduction	19 (19.4)	22 (22.4)	0.598
NCI-CTC grade 1 †	17 (17.3)	21 (21.4)	
NCI-CTC grade 2 ‡	2 (2.0)	1 (1.0)	
NCI-CTC grade 3 ^*^	0	0	
NCI-CTC grade 4⁑	0	0	
Cardiac death	0	0	

LVEF, left ventricular ejection fraction; NCI-CTC, National Cancer Institute common toxicity criteria.

Data are n (%). †Asymptomatic reduction of 10% or more, but less than 20% of baseline. ‡Asymptomatic reduction to lower limit of normal or 20% or more of baseline. ^*^Congestive heart failure, responsive to treatment. ⁑Refractory or poorly controlled heart failure due to drop in ejection fraction. Lower limit of normal:EF value = 50%.

### Disease-free survival and overall survival

**Figure 3:**
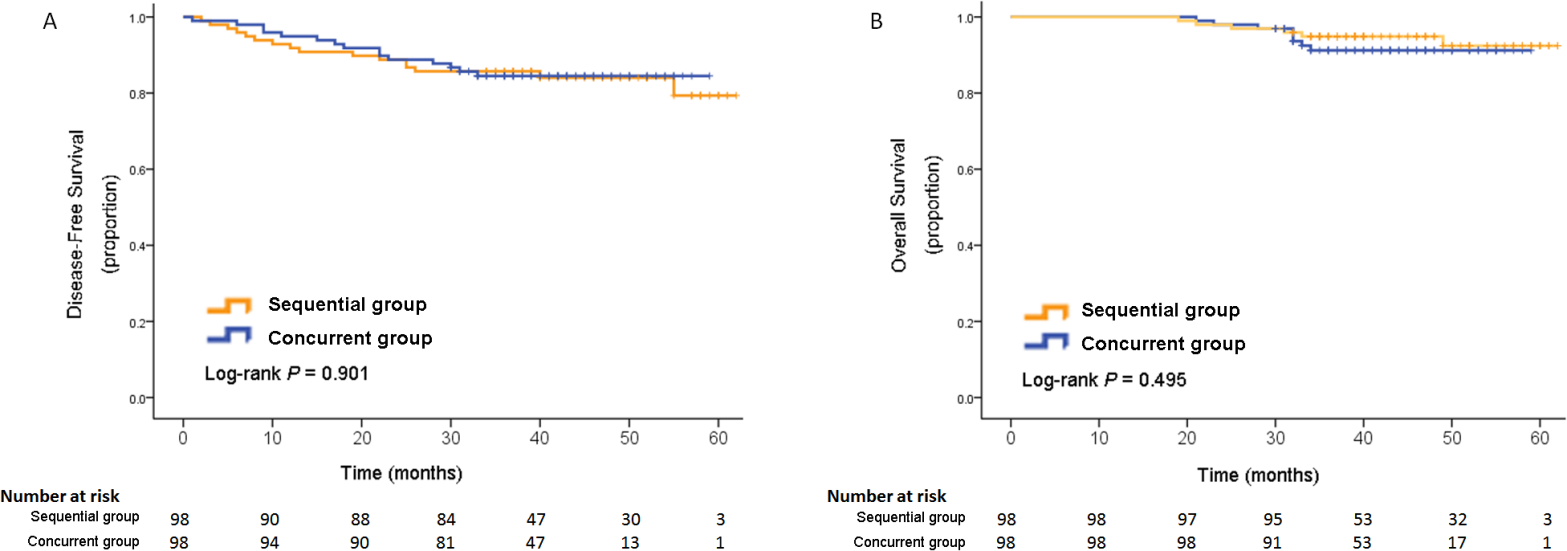
Kaplan-Meier estimates of disease-free survival **(A)** and overall survival **(B)**.

Fifteen recurrent events were observed in the concurrent group and 16 events in the sequential group (Table 3 and Figure 3). The percentages of patients alive and disease-free at five years were 84.5% in the concurrent group and 79.4% in the sequential group (hazard ratio, 0.956; 95% CI 0.471–1.939; *P* = 0.901). There was no significant difference between the rates of DFS in the two groups.

**Table 3:** First disease-free survival events by trial arms

	Concurrent group(N=98)	Sequential group(N=98)
Locoregional recurrence	4	3
Chest wall	1	0
Lymph nodes	3	3
Distant metastasis	12	13
Bone	4	7
Brain	4	1
Liver	1	3
Lung	3	2
Total DFS events	15^*^	16

^*^ One case presented with both bone and lymph nodes involvement as first manifestation of recurrence.

There were 14 deaths occurred during follow-up, eight in the concurrent group and six in the sequential group. The Kaplan-Meier estimates of the OS at five years was 91.2% in the concurrent group and 92.4% in the sequential group (hazard ratio, 1.443; 95% CI 0.500–4.167; *P* = 0.495; Figure 3). The difference was also non-significant.

## DISCUSSION

To our knowledge, this is the first randomised and controlled trial that demonstrated the safety of the concurrent use of trastuzumab and anthracyclines in the adjuvant treatment of early breast cancer. With a median follow-up time of 42 months, 19.4% of the patients in the concurrent group and 22.4% in the sequential group met the criteria for cardiac events. There was no patient documented congestive heart failure or cardiac death. No significant difference was found in cardiac safety event rates between the two groups (*P* = 0.598). A slight decline in the mean LVEF values was observed during the administration of trastuzumab; however, they recovered in both groups within one year.

Although the concurrent use of trastuzumab and anthracyclines caused unacceptably high rates of cardiac dysfunction in the metastatic setting [7], this regimen was proven fairly safe in the neoadjuvant setting [8, 9]. No prospective randomised trial to investigate the cardiac safety of the concurrent regimen of trastuzumab and anthracyclines in the adjuvant treatment of early breast cancer has been reported before. Only one Japanese study by Watanabe and colleagues [14, 15] retrospectively reviewed the cardiac tolerability of the concurrent administration of trastuzumab and anthracyclines. In that analysis, 49 patients with HER2-positive breast cancer received paclitaxel (P) followed by epirubicin, fluorouracil, and cyclophosphamide (FEC) and concurrent trastuzumab (Trastuzumab Group). The LVEF values of the patients in the Trastuzumab Group was assessed regularly for more than one year and compared with those of HER2-negative breast cancer patients who received P followed by FEC (Standard Group). Twelve patients (24.9%) in the Trastuzumab Group had a LVEF reduction of more than 10%, compared to 22.2% in the Standard Group [14, 15]. There was no significant difference in LVEF reduction between the two groups (*P* = 0.386) [14, 15]. The cardiac event rate of this retrospective study was similar to that of our trial, which supported our conclusion regarding the cardiac safety of the concurrent administration of trastuzumab and anthracyclines.

Nevertheless, it is unclear whether there is anthracycline-trastuzumab interaction contributing to cardiotoxicity, although some possible mechanisms have been proposed [16, 17]. It is currently considered that anthracyclines cause type I cardiac damage, which is dose-related and associated with identifiable ultrastructural abnormalities, and begins from the initial administration of the drug [18]. While trastuzumab-related cardiotoxicity is classified as Type II, which is highly reversible, non-dose-dependent, and unassociated with ultrastructural changes [18]. Since the cardiac toxicity caused by anthracyclines persists during the sequential use of trastuzumab, we propose that the timing of trastuzumab administration is not related to the severity of cardiotoxicity. The high rate of cardiac failure in the metastatic setting might not be caused by the concurrent regimen of trastuzumab and anthracyclines, but by the high cumulative dose of anthracyclines and poor baseline cardiac conditions of the metastatic patients. Therefore, there suggested a high possibility the cardiotoxicity rate would not change even trastuzumab was given sequentially in that trial. The absence of observations of a high rate of cardiotoxicity in all other related trials also provides evidence in support of our interpretation.

Apart from the well-tolerated concurrent administration of trastuzumab and anthracyclines in the adjuvant treatment of breast cancer, there are at least two other reasons to prefer this regimen.

Firstly, the required treatment duration is the shortest of all regimens containing both trastuzumab and anthracyclines, and all phases of this regimen can be completed within 12 months. This will shorten the duration of treatment by 3–6 months, and reduce considerably hospitalization times and costs.

Secondly, the genes encoding topoisomerase II alpha and HER2, the targets for anthracyclines and trastuzumab, respectively, are frequently co-amplified in breast cancers [19]. Pre-clinical studies showed there were additive and/or synergistic therapeutic effects between anthracyclines and trastuzumab [20]. The findings of some trials also suggested that the HER2 status was a predictor of responsiveness to adjuvant anthracyclines administration, and patients with HER2-positive breast tumor might derive preferential benefit from the administration of anthracyclines [4–6]. The concurrent administration of trastuzumab and anthracyclines in neoadjuvant trials has resulted in high rates of pCR and prolonged survival [8–10].

One limitation of our trial is the relatively small number of patients to show the secondary endpoint of survival benefits. However, the safety issue has priority over the effect, and for more than a decade, cardiac safety has been the obstacle that prevented clinical researchers from investigating the effect of the concurrent administration of trastuzumab and anthracyclines in the adjuvant treatment of breast cancer. The present trial has provided the first evidence of cardiac safety, but larger trials are warranted to confirm the effect.

Concurrent administration of trastuzumab and anthracyclines is a safe adjuvant regimen with a low frequency of cardiac dysfunction, although careful cardiac monitoring is needed. Due to the limitation of the relatively small sample size and short follow-up time, further multicenter clinical trials are required to determine the cardiac safety of the concurrent regimen and whether it improves the survival of patients.

## MATERIALS AND METHODS

### Study design

The present study was a prospective, randomised, and controlled trial in patients with newly diagnosed early breast cancer from Peking Union Medical College Hospital (PUMCH). At the time of registration, the patients with histopathologically confirmed Her2-positive early breast cancer had undergone definitive surgery and planned to receive anthracycline-based adjuvant chemotherapy and trastuzumab treatment. The primary study endpoint was cardiac safety, including symptoms, electrocardiogram, and LVEF changes. Secondary endpoints involved DFS and OS. DFS was defined as the interval from randomization to: local, regional, or distant recurrence; second primary cancer; contralateral breast cancer; or death from any cause, whichever came first. OS was defined as the time from random assignment to death from any cause.

The study was approved by the Institutional Review Board of PUMCH. All patients provided written informed consent. The study was conducted in accordance with the Declaration of Helsinki.

This study was registered at the ClinicalTrials.gov (Identifier: NCT01413828).

### Eligibility criteria

Eligibility requirements included primary operable node-positive or high-risk node-negative invasive breast cancer. All tumors had to be removed by definitive surgery within 60 days of trial registration, and neoadjuvant treatments were not permitted.

All the patients had HER2-positive breast cancer, which was defined as immunohistochemistry (IHC) score of 3+ (Herceptest, Dako, Denmark) or gene amplified by fluorescence *in situ* hybridization (FISH, Pathvysion HER2 test, Abbott-Vysis, USA) according to the PUMCH laboratory. Participants were excluded if they had locally advanced or metastatic breast cancer, bilateral breast cancer, previous or concurrent any other malignant disease, previous administration of anthracyclines and/or trastuzumab for any disease.

Only adult female patients with adequate hepatic, renal, and bone marrow functions were included as participants in the investigation. An additional requirement for enrollment included having a LVEF value of 55% or higher measured by echocardiography. Patients were ineligible if they had a history of documented congestive heart failure, angina pectoris, unstable arrhythmias, coronary artery disease with previous myocardial infarction, clinically significant valvular disease, and uncontrolled hypertension.

### Randomisation and masking

Eligible participants were randomly assigned, in a 1:1 ratio, to receive adjuvant therapy with trastuzumab administered concurrently with anthracyclines (concurrent group) or sequentially to anthracyclines (sequential group). The chemotherapy regimen and radiation therapy was chosen before randomisation. Randomisation was done by a computer-generated randomization sequence, using a permuted-block randomisation method with a block size of four.

### Treatment regimens

Eligible participants were randomised after definitive surgery to receive concurrent or sequential administration of trastuzumab and anthracyclines. The regimens of chemotherapy consisting of at least four cycles of anthracycline-based treatment were chosen at the discretion of their physician based on the National Comprehensive Cancer Network guidelines before randomisation. If anthracyclines were given alone or followed by taxanes, the starting doses of doxorubicin and epirubicin were 60 mg/m^2^ and 90–100 mg/m^2^, respectively. Alternatively, if anthracyclines were administered concurrently with taxanes, doses of 50 mg/m^2^ of doxorubicin and 75 mg/m^2^ of epirubicin were employed. In all cases, the maximum cumulative allowable doses were 360 mg/m^2^ of doxorubicin and 600 mg/m^2^ of epirubicin. The anthracyclines were given every two or three weeks.

Trastuzumab was administered intravenously, beginning with a loading dose of 8 mg/kg of body weight, and followed by maintenance doses of 6 mg/kg given every three weeks for one year. If the treatment of trastuzumab was delayed by more than one week, the administration was restarted with the initial dose of 8 mg/kg, followed by the usual maintenance dose (6 mg/kg every three weeks).

Radiation therapy was required after completion of chemotherapy to patients who underwent breast-conserving surgery or mastectomy with at least four positive nodes. Patients with hormone receptor-positive disease were given adjuvant endocrine therapy after chemotherapy unless contraindicated. Trastuzumab treatment was continued during radiotherapy and/or endocrine therapy. No cardioprotective drugs (dexrazoxane, angiotensin-converting enzyme inhibitors, beta blockers, et al.) were permitted to use prophylactically.

### Cardiac safety

In addition to monitoring general safety, an intense cardiac monitoring was also scheduled. Cardiac events were graded according to the National Cancer Institute Common Toxicity Criteria, version 2.0 (NCI-CTC, version 2.0). Cardiac monitoring in the concurrent group and the sequential group included history-taking, a 12-lead electrocardiogram, and an assessment of LVEF by echocardiography at baseline and at 3, 6, 9, 12, and 24 months after the first dose of trastuzumab. Thereafter, history-taking and physical examination were scheduled every 6 months until the end of the study. Additional scans could be performed at the investigator's discretion. Cardiac safety was monitored by independent cardiologists.

If the absolute value of LVEF declined by 20% or more from baseline or below 50%, trastuzumab was temporarily discontinued for four weeks during which the LVEF value was reassessed. The administration of trastuzumab was permanently discontinued in case that LVEF values remained below these levels or the participant experienced symptomatic cardiac failure.

### Statistical design and analysis

In the present trial, the primary end point was cardiac safety, defined as the incidence of an asymptomatic LVEF reduction of 10% absolutely or more from baseline, or below 50%, or symptomatic heart failure. To calculate the sample size, we had to assume the rate of cardiac safety events in each of the two groups, whose values can vary due to the different definitions and populations used in each specific trial. We adopted the rate of cardiac dysfunction of the Breast Cancer International Research Group 006 trial, in which 18.6% of participants in the sequential group had a LVEF reduction of more than 10% [3]. According to a trial in the metastatic setting, in which prohibition of the concurrent administration of trastuzumab and anthracyclines in clinical practice was recommended, the rate of heart failure in the concurrent group was increased to 27% [7]. Thus, we can infer a much higher rate of asymptomatic LVEF reduction of the concurrent administration of trastuzumab and anthracyclines since asymptomatic LVEF reduction is much more common than heart failure. In our investigation, if we were to assume rates of cardiac events in the sequential group and the concurrent group of 19% and 32%, respectively, 176 participants would have been needed to achieve a two-sided statistical significance of 5% with 80% power.

Categorical data were compared using the two-tailed chi-square test. When the expected counts were low, we employed the Fisher's exact test. Quantitative data were compared by Student's *t*-test. The DFS and OS were estimated using the Kaplan-Meier method. Two-sided log-rank test for time-to event endpoints were used. Differences were considered significant at *P* < 0.05.
